# Towards Standardized Assessment of Outcomes in Back Pain—Validation of Linking Studies Between Disease-Specific and Generic Patient-Reported Outcome Measures

**DOI:** 10.3390/jcm13216524

**Published:** 2024-10-30

**Authors:** Claudia Hartmann, Gregor Liegl, Matthias Rose, Felix Fischer

**Affiliations:** 1 Center for Patient-Centered Outcomes Research, Charité—Universitätsmedizin Berlin, Corporate Member of Freie Universität Berlin and Humboldt-Universität zu Berlin, 10117 Berlin, Germanyfelix.fischer@charite.de (F.F.); 2 Department of Psychosomatic Medicine, Charité—Universitätsmedizin Berlin, Corporate Member of Freie Universität Berlin and Humboldt-Universität zu Berlin, 10117 Berlin, Germany

**Keywords:** Oswestry Disability Index (ODI), PROMIS Profile 29, linking, standardization, patient-reported outcome measures

## Abstract

**Background**: Comparing outcomes across different health measurement tools is essential where various patient-reported outcome measures (PROMs) are used. In spinal surgery, where recent studies show that over 30 different PROMs are applied, this need becomes even more pressing. Although several statistical transformations between the Oswestry Disability Index (ODI) and the PROMIS Profile 29 have been proposed, validation studies on conversion equations and cross-walk tables remain limited. In this study, we examined the agreement between observed ODI scores and those predicted from the PROMIS Profile 29 in a large sample of patients with low back pain, collected from routine clinical care. **Methods**: We compared the performance of regression and linking models at both the individual and group levels. Using Bland–Altman plots, we assessed the mean difference, 95% limits of agreement, root mean squared error (RMSE), and standardized mean differences (Cohen’s d) between predicted and observed ODI scores. **Results**: While group-level agreement was satisfactory, with negligible effect sizes, individual prediction accuracy was relatively poor. Additionally, regression models showed inconsistent performance across the ODI score range, though incorporating more domains marginally improved predictions. **Conclusions**: The equipercentile linking approach demonstrated stable agreement across all ODI scores, making it the preferred method. Future regression models should account for nonlinear relationships between PROMs to enhance prediction accuracy.

## 1. Introduction

The collection of Patient-Reported Outcomes (PROs) to assess measurable and actionable predictors of health-related quality of life (HRQOL) is well established in research on spinal injuries [1]. Recently, gathering the patient’s perspective on treatment outcomes has become increasingly common in routine care as well [2]. This shift is driven by a move towards patient-centered healthcare and the aim to better understand the trajectory of the healing process, as well as to compare outcomes across different treatments [1,3,4].

This interest has led to the development of many different Patient-Reported Outcome Measures (PROMs) over the past decades. Over 30 spine-specific PROMs were identified in a systematic literature review in 2022, with the Oswestry Disability Index (ODI) and Neck Disability Index (NDI), followed by the Roland-Morris Disability Questionnaire (RMDQ), being the most frequently used PROMs [5]. More recently, generic PROMs such as the EQ-5D [6] or the PROMIS Profile 29 [7] have been used to assess PROs in patients with various conditions, including spinal injuries and back pain [8,9,10]. Unfortunately, this plethora of different PROMs introduces challenges. As early as 1998, a systematic literature review by Bhashyam et al. concluded that due to variations in scales and transformation methods, HRQOL results for the treatment of traumatic musculoskeletal injuries were not comparable, and meta-analyses could not be performed [11].

Given that the available PROMs in spinal surgery come with specific scales, it is important to develop methods to enable quantitative comparisons across measures. When looking at the most relevant spine-specific PROMs it becomes evident that they have a large overlap in content, focusing merely on pain, functional limitations such as walking and standing, or different aspects of personal care. Differences are seen in the number of items and the variety of functional limitations assessed [12]. To address the issue of comparability, three primary approaches have been proposed.

One method for ensuring comparability is to establish a standard set of outcome measures [13]. A variety of standard sets have been established for a number of diseases. [14,15]. These sets frequently include a core set of PRO measures (PROMs) that have been demonstrated to be well-validated, reliable, and sensitive to change. This approach has been pursued by several international and national initiatives, such as the International Consortium for Health Outcomes Measurement (ICHOM) and the Core Outcome Measures in Effectiveness Trials (COMET) Initiative. ICHOM developed a set for lower back pain [16] and COMET lists seven Core Outcome Set studies for low back pain [17]. The main disadvantages of this approach are the variety in standards, the lack of flexibility to incorporate new measurement methods, and the loss of comparability with existing instruments, as well as the inability to adapt to changes in licensing agreements [18]. Furthermore, this approach does not enable the comparison of existing data.

One approach frequently used to address this is the development of direct conversion methods using statistical techniques such as regression analysis [19]. These methods are designed to enable the translation of scores, for example, between different PROMs, through the use of conversion equations, and they have been developed for the ODI and the PROMIS Profile 29 by Pennings et al. [20] and Edelen et al. [21]. The use of linear regression models allows for the simultaneous inclusion of multiple predictors, thereby offering flexibility. Moreover, the coefficients generated by these models are straightforward to interpret, facilitating a more comprehensive understanding of the contribution of each variable to the overall outcome. A key limitation of the regression approach is that it is only capable of making one-way predictions, in contrast to the ability of the common metric to accommodate bidirectional predictions. A further limitation is the sensitivity to outliers, which can distort the agreement of the model and lead to unreliable results. Furthermore, if the independent variables are highly correlated (multicollinearity), the model’s coefficients may become unstable, thereby hindering the accurate interpretation of the impact of each variable on the outcome [22]. In spite of these limitations, their overall simplicity and ease of implementation make regression-based conversion methods a practical tool in a wide range of clinical settings.

A different approach is linking different PROMs assessing the same or similar outcomes to a common metric [23]. This approach allows for the direct comparison of scores from different instruments, facilitating data pooling and meta-analysis. The PROMIS T-score metric, based on item response theory, is an example of a common metric to which several instruments have been linked [24,25,26]. Overviews of linked PROMs can be found on the PROsetta Stone^®^ website (www.prosettastone.org, accessed on 29 October 2024) or on common-metrics.org, accessed on 29 October 2024 [27]. A principal benefit of this approach is the creation of linking tables that enable the comparison of scores on both scales. A variety of linking methods exist, including equipercentile linking and IRT-based linking methods [23]. This approach requires that linked measures address the same underlying construct. Moreover, the development of reliable linking methods requires the use of large and representative datasets. In the absence of adequate data, the linking equations may lack robustness and fail to provide accurate conversions.

Despite the fact that several statistical transformations between ODI and the PROMIS Profile 29 have been proposed, validation studies of both conversion equations and cross-walk tables in independent data are scarce. Hence, we investigated the agreement between observed ODI scores and ODI scores predicted using the PROMIS Profile 29 in a large sample of patients suffering from low back pain, collected during routine clinical care. We were particularly interested in comparing the performance of regression and linking models at both individual and group levels.

## 2. Materials and Methods

### 2.1. Sample

ODI and PROMIS-29 Profile data were digitally collected at the first visit, before treatment, from all patients attending the Spine Centre of the Charité—Universitaetsmedizin Berlin. We used the translated and validated versions of the measures in the German language [28,29]. For our analysis, we included the first PRO assessment of patients aged over 18 years with lumbar or thoracic spine complaints during the period of April 2019 to February 2024. Patients with neck pain or problems in the cervical spine were excluded. Overall, 1073 patients met the criteria. As the digital survey restricts the skipping of items, no item responses were missing.

### 2.2. Measures

The disease-specific ODI is the most used PROM for lumbar spine disease [5] and was first published in 1980 as a 10-item instrument developed specifically to quantify the disability caused by low back pain [30]. It is based on classical test theory (CCT) and focuses on pain intensity and pain’s interference with daily activities, such as personal hygiene, walking, lifting, sitting, and standing. Additionally, aspects of social roles and activities such as travel are covered. Each item is rated on a six-point scale, with individual response options for each item. Response number 1 reflects no interference by pain during the activity, while response number 6 reflects the highest level of pain interference. The instrument has no recall period [31].

The generic PROMIS-29 Profile is part of the Patient-Reported Outcomes Measurement Information System (PROMIS) and is based on item response theory. The Profile is a 29-item questionnaire that uses 4-item short forms to assess seven core constructs of HRQOL: pain interference (PI), depression (DE), fatigue (FA), anxiety (AN), physical function (PF), sleep disturbance (SD), and ability to participate in social roles and activities (SRAA). The instrument utilizes a five-point Likert scale, with the exception of the domain of pain intensity (PIN), which is measured on a ten-point visual analogue scale (VAS). The three scales of PI, SD, and FA employ a five-point Likert scale, with 5 indicating “Not at all” and 1 indicating “Very much”. In contrast, the AN, DE, and SRAA scales utilize a five-point scale ranging from 5 “Never” to 1 “Always”. The PF domain employs a Likert scale that ranges from 5 “Without any difficulty” to 1 “Unable to do”. The recall period is seven days, with the exception of the PF and SRAA domains, which do not have a specified recall period. A T-score is calculated for each domain, where 50 is the mean and 10 is the standard deviation of the US general population [7,32].

### 2.3. Statistical Analysis

We report the descriptive variables of the sample using frequencies for categorical variables and mean and standard deviation for continuous variables. We also report the correlations between the PROMIS Profile 29 domains and the ODI score.

Several proposals have been made to translate scores between the ODI and the PROMIS-29 Profile. Pennings et al. [20] estimated a conversion equation, using 719 patients undergoing spine surgery, to predict ODI sum scores based on six PROMIS 29 domain T-Scores. Pennings 1:

ODI = 36.369 − 0.739 × PROMIS Physical Function + 2.026 × PROMIS Pain Intensity +  0.231 × PROMIS Sleep Disturbance − 0.340 × PROMIS Social Participation + 0.223 × PROMIS Pain Interference + 0.135 × PROMIS Depression.

Pennings et al. [20] also reported an equation which estimates the ODI score based on the PROMIS Physical Function T-Score alone. Pennings 2:

ODI = 109.129 − 1.920 × PROMIS Physical Function.

More recently, Edelen et al. [21] also developed three regression models to predict ODI sum scores from PROMIS Physical Function and Pain Interference T-Scores, as well as Pain Intensity ratings, and vice versa. We used the model with the combined sample of 1736 measurements were PROMIS measures were included and patients were treated for chronic low back pain. Edelen:

ODI = 17.572−0.789 × PROMIS PF + 0.623 × PROMIS Pain Interference + 1.192 × PROMIS Pain Intensity.

Tang et al. [33] used equipercentile linking to provide a cross-walk table between PROMIS Pain Interference and ODI scores. We applied a linear approximation to interpolate the predicted ODI scores corresponding to PROMIS T-Scores, refined to one decimal place.

The aim of this study is to validate these prediction algorithms using independent data. Therefore, we applied the transformation formulas’ respective cross-walk tables and compared the predicted and observed ODI scores using Bland–Altman plots. We also calculated the mean difference between predicted and observed ODI scores, estimated the 95% limits of agreement, and reported the root mean squared error (RMSE) as well as the standardized mean differences (Cohen’s d) between observed and predicted ODI scores to enable model comparisons. As recommended by Cohen, d values smaller than 0.2 were interpreted as negligible effects, while values of 0.2, =0.5, and 0.8 were interpreted as small, medium, and large effect sizes, respectively [34].

## 3. Results

### 3.1. Baseline Characteristics

The mean age of the sample is 56.9 years (SD = 17.5 years, range 18–95), and 53.3% of the patients included are women. Back pain has been present for 3–12 months in 22.1% of respondents, for 1–2 years in 12.1%, and for more than 2 years in 40.4%. Compared to the general population (mean score 50 ± 10), respondents report higher-than-average pain interference (63.2) and lower-than-average physical functioning (37.8), with a mean pain intensity score of 6.12. For 51%, the ODI score reports a severe disability or higher, 29% score a moderate disability, and 19% a mild disability. The average ODI score of the sample is 41.2 (for further details, see Table 1).

The ODI correlates highly (>0.6) with the PROMIS-29 domains PF, PI, PIN, and SAA. As expected, the highest correlations (−0.8 and 0.81) are found for the PF and PI domains. The PROMIS-29 domains FA, SL, and DP range between 0.43 and 0.45, indicating a moderate correlation, while the domain AN shows a low correlation with a coefficient of 0.36. All coefficients are summarized in Table 2.

### 3.2. Validationp

**Figure 1:**
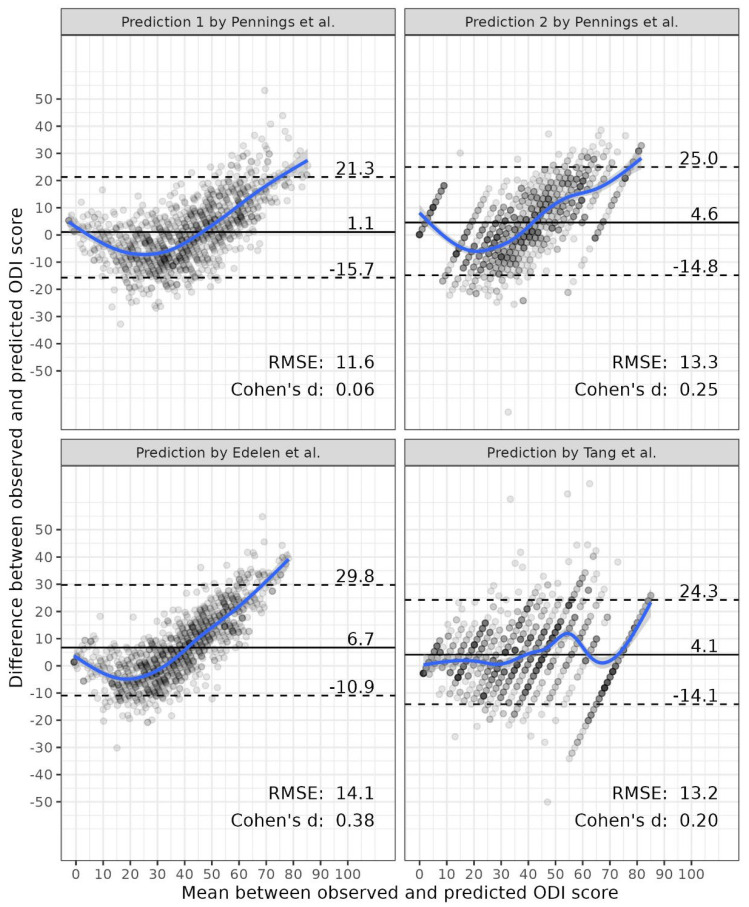
Bland-Altman plots of observed and predicted scores [20,21,33].

Figure 1 shows the agreement between the observed ODI scores and the respective predictions by Pennings et al., Edelen et al., and Tang et al. [20,21,33]. We find that the prediction model including six PROMIS domains used by Pennings et al. [20] performs best in terms of bias (1.1 ODI points) and average error (RMSE = 11.6). At the group level, the effect size of the difference between the observed and predicted ODI scores is negligible (d = 0.06). However, the precision of individual predictions compared to actual observed scores is poor, with 90% of the differences falling within an interval between −15.7 and 21.3 points.

Despite negligible to small overall bias, all regression-based prediction models show systematic deviations from the linear prediction—higher ODI scores are consistently underestimated by the respective predictions. This suggests that linear prediction models do not perform equally well across the continuum of ODI scores and suffer from regression to the mean. In contrast, the prediction of ODI scores based on equipercentile linking shows a more consistent association between observed and predicted scores across the score continuum, although the bias and precision are similar to that of the regression-based approaches. Furthermore, our results indicate that models incorporating only one PROMIS domain [20,33] suffer from a ceiling effect, where variation in the ODI is not captured by the PROMIS domain scores derived from the four-item short forms used in this study.

## 4. Discussion

We investigated the agreement of four different models in the prediction of ODI scores using domains from the PROMIS Profile 29 in a large, independent dataset of spine patients who completed both the ODI and the PROMIS Profile 29 forms. At the group level, the agreement was deemed satisfactory, with negligible to small effect sizes observed between predicted and actual ODI scores. However, our results indicate that the precision of individual predictions was relatively poor. Additionally, the regression-based models did not perform equally well across the range of ODI scores, and the inclusion of more domains in the model resulted in a slightly improved performance.

In our study, the discrepancy between predicted and observed scores at the individual level was high, which has been described repeatedly in previous linking studies [26,35]. However, it is important to consider to what extent this imprecision is a result of the linking procedure, or the inherent imprecision of PROMs itself. Assuming a reliability of 0.90, we would expect the limit of agreement between two consecutive ODI measurements to be about 16 points, assuming that a reliability of 0.85 would equal about 19 points. This shows that only a small fraction of individual prediction error can be explained by the actual linking.

On a group level, we observed biases between ODI scores of 1.1 and 6.7, corresponding to negligible to small effect sizes. The relevance of this bias depends on applications. While it may be too large to investigate small effects between groups assessed with different instruments, it could still be justifiable to compare discrepancies across studies or guide general interpretations.

Regression-based conversion equations showed that low observed ODI values were overestimated, and high scores were underestimated. This can be explained due to the nonlinear relationship between the scores, which is not accounted for by linear regression. Utilizing more complex regression models incorporating splines or polynomials have the potential to address these problems, but will yield more complex conversion equations.

Better results were observed with the cross-walk table based on equipercentile linkage, modelling the empirical relationship between the data rather than a linear one. Still, we observed an overall bias (negligible-to-small effect size) comparable to that of the regression-based models. In this regard, our findings indicate that the model including six PROMIS domains yields the lowest bias across all models and that models including single domains might suffer from ceiling effects when only a few items are used. This shows that additional information might improve prediction and that regression models have applications where linking-based cross-walk tables cannot be used.

This is a validation study of prediction models using independent data and, therefore, a true test of the performance of these models. Since the data are derived from routine care, the study has a high degree of external validity. A further strength of this study is that we compared four models with different methodological approaches. Nonetheless, the following limitations need to be considered. First, the prediction models were developed using English-speaking samples in the US, whereas we collected data during routine care in Germany. Hence, there are likely systematic differences between the questionnaires, patients, and health systems, contributing to the less-than-perfect performance of the prediction models.

In addition to the aforementioned effects, differences in the respective samples could also affect our findings. The sample sizes with which the prediction models were used differ from N = 719 [20] to N = 1.736 [21] and N = 9.020 [33]. It is important to consider that the prediction models performing estimations of smaller samples might yield less accurate results. Furthermore, there are significant differences in the specific PROs between the samples. ODI scores vary from 22.4 [21] to 34.4 [33] and 45.4 [20], indicating that prediction models were used to make estimates of samples differing in actual symptom burden. This is also reflected in the PROMIS PIN scores (4.2., 6.0, and 6.8). The other relevant PROMIS scores, PF and PI, do not reflect such differences, as Edelen et al. [21] reported a PI score of 56.3 compared to Tang et al. [33] 62.3, Pennings et al. [20] 66.3, and the 62.6 from our sample. However, correlations between samples were similar. The correlation coefficients between ODI and PROMIS reported by Penning et al. show very similar correlations (≤0.1) to the relevant domains PF, PI, and SAA, which is also true for the data from Edelen et al. [21] (N = 1.895) and the PROMIS PI used in the linking study by Tang et al. [33].

## 5. Conclusions

We investigated the agreement of several published models in the prediction of ODI scores, based on one or more scales from the PROMIS Profile 29 and using an independent sample of patients with low back pain. Our findings indicate that, while these models performed sub-optimally in predicting individual ODI scores, the discrepancies between observed and predicted scores were negligible at the group level. The equipercentile linking approach demonstrated stable agreement across the full range of ODI scores, making it the most recommended method. Regarding regression-based approaches, our data suggest that future models should account for nonlinear relationships between different PROMs to improve prediction accuracy.

## Figures and Tables

**Table 1:** Sample baseline demographics and HRQOL characteristics (n = 1073).

	n (%)
Demographics	
Age, Mean (SD)	56.9 (17.5)
Age, Range	18–95
Age Group	
<30	101 (9.4%)
30–40	130 (12.1%)
41–50	134 (12.5%)
51–60	215 (20.0%)
61–70	210 (19.6%)
>70	283 (26.4%)
Education, College or Higher	430 (40%)
Employed	369 (34.4%)
Retiree	365 (34.0%)
HRQOL	n (SD)
ODI	41.2 (21.7)
PROMIS PF	37.8 (7.7)
PROMIS PI	63.2 (8.0)
PROMIS PIN	6.1 (2.4)
PROMIS FA	53.8 (10.0)
PROMIS SL	53.6 (8.8)
PROMIS AN	55.4 (9.8)
PROMIS DP	54.6 (9.4)
PROMIS SAA	42.9 (9.3)

**Table 2:** Correlation between ODI and PROMIS-29.

		ODI	PROMIS						
			PF	PI	PIN	FA	SL	AN	DP
PROMIS	PF	**−0.83**							
	PI	**0.81**	**−0.79**						
	PIN	**0.65**	−0.58	**0.69**					
	FA	0.45	−0.40	0.51	0.42				
	SL	0.44	−0.35	0.45	0.43	0.56			
	AN	0.36	−0.31	0.40	0.33	0.59	0.43		
	DP	0.43	−0.37	0.45	0.36	**0.68**	0.47	**0.75**	
	SAA	**−0.73**	0.71	**−0.81**	−0.54	−0.52	−0.40	−0.39	−0.46

Bold: high correlations.

## Data Availability

Data can be shared on request to the main author.

## References

[1] Young K., Gang C.H., Vaishnav A.S., Jivanelli B., Steinhaus M.E., Lovecchio F.C., Qureshi S.A., McAnany S.J., Kim H.J., Iyer S. (2019). The use of patient-reported outcomes measurement information system (PROMIS) in spine surgery: A systematic review. Spine J..

[2] Black N. (2013). Patient reported outcome measures could help transform healthcare. BMJ.

[3] Hambrecht J., Köhli P., Chiapparelli E., Amoroso K., Lan R., Guven A.E., Evangelisti G., Burkhard M.D., Tsuchiya K., Duculan R. (2024). The Disaggregation of the Oswestry Disability Index in Patients undergoing Lumbar Surgery for Degenerative Lumbar Spondylolisthesis. Spine J..

[4] Jacob K.C., Patel M.R., Parsons A.W., Vanjani N.N., Pawlowski H., Prabhu M.C., Singh K. (2021). The Effect of the Severity of Preoperative Back Pain on Patient-Reported Outcomes, Recovery Ratios, and Patient Satisfaction Following Minimally Invasive Transforaminal Lumbar Interbody Fusion (MIS-TLIF). World Neurosurg..

[5] Beighley A., Zhang A., Huang B., Carr C., Mathkour M., Werner C., Scullen T., Kilgore M.D., Maulucci C.M., Dallapiazza R.F. (2022). Patient-reported outcome measures in spine surgery: A systematic review. J. Craniovertebr. Junction Spine.

[6] (1990). EuroQol—A new facility for the measurement of health-related quality of life. Health Policy.

[7] Cella D., Choi S.W., Condon D.M., Schalet B., Hays R.D., Rothrock N.E., Yount S., Cook K.F., Gershon R.C., Amtmann D. (2019). PROMIS^®^ Adult Health Profiles: Efficient Short-Form Measures of Seven Health Domains. Value Health.

[8] Carreon L.Y., Djurasovic M., Dimar J.R., Owens R.K., Crawford C.H., Puno R.M., Bratcher K.R., McGraw K.E., Glassman S.D. (2016). Can the anxiety domain of EQ-5D and mental health items from SF-36 help predict outcomes after surgery for lumbar degenerative disorders?. J. Neurosurg. Spine.

[9] Lehr A.M., Delawi D., van Susante J.L.C., Verschoor N., Wolterbeek N., Oner F.C., Kruyt M.C. (2021). Long-term (>10 years) clinical outcomes of instrumented posterolateral fusion for spondylolisthesis. Eur. Spine J..

[10] Khutok K., Janwantanakul P., Jensen M.P., Kanlayanaphotporn R. (2021). Responsiveness of the PROMIS-29 Scales in Individuals with Chronic Low Back Pain. Spine.

[11] Bhashyam A.R., van der Vliet Q.M.J., Ochen Y., Heng M., Leenen L.P.H., Hietbrink F., Houwert R.M. (2019). Injury-related variation in patient-reported outcome after musculoskeletal trauma: A systematic review. Eur. J. Trauma. Emerg. Surg..

[12] Chmielewski B., Wilski M. (2024). Psychometric Properties of Chosen Scales Evaluating Disability in Low Back Pain—Narrative Review. Healthcare.

[13] Kirkham J.J., Williamson P. (2022). Core outcome sets in medical research. BMJ Med..

[14] ICHOM Sets. https://www.ichom.org/patient-centered-outcome-measures/.

[15] COMET Database. https://www.comet-initiative.org/Studies.

[16] Clement R.C., Welander A., Stowell C., Cha T.D., Chen J.L., Davies M., Fairbank J.C., Foley K.T., Gehrchen M., Hagg O. (2015). A proposed set of metrics for standardized outcome reporting in the management of low back pain. Acta Orthop..

[17] COMET Core Outcome Set Low Back Pain. http://www.comet-initiative.org/studies/searchresults?guid=e9f7d294-b25b-45b3-a2e4-269469b1a52a.

[18] Fried E.I.E.d.B. (2021). From mandating common measures to mandating common metrics: A plea to harmonize measurement results. PsyArXiv.

[19] Liegl G., Fischer F.H., Martin C.N., Rönnefarth M., Blumrich A., Ahmadi M., Boldt L.-H., Eckardt K.-U., Endres M., Edelmann F. (2024). Converting PROMIS^®^-29 v2.0 profile data to SF-36 physical and mental component summary scores in patients with cardiovascular disorders. Health Qual. Life Outcomes.

[20] Pennings J.S., Devin C.J., Khan I., Bydon M., Asher A.L., Archer K.R. (2019). Prediction of Oswestry Disability Index (ODI) using PROMIS-29 in a national sample of lumbar spine surgery patients. Qual. Life Res..

[21] Edelen M.O., Rodriguez A., Herman P., Hays R.D. (2021). Crosswalking the Patient-Reported Outcomes Measurement Information System Physical Function, Pain Interference, and Pain Intensity Scores to the Roland-Morris Disability Questionnaire and the Oswestry Disability Index. Arch. Phys. Med. Rehabil..

[22] Vatcheva K.P., Lee M., McCormick J.B., Rahbar M.H. (2016). Multicollinearity in Regression Analyses Conducted in Epidemiologic Studies. Epidemiology.

[23] Schalet B.D., Lim S., Cella D., Choi S.W. (2021). Linking Scores with Patient-Reported Health Outcome Instruments: A Validation Study and Comparison of Three Linking Methods. Psychometrika.

[24] Schalet B.D., Revicki D.A., Cook K.F., Krishnan E., Fries J.F., Cella D. (2015). Establishing a Common Metric for Physical Function: Linking the HAQ-DI and SF-36 PF Subscale to PROMIS^®^ Physical Function. J. Gen. Intern. Med..

[25] Liegl G., Fischer F.H., Woodward M., Török M., Strippoli G.F.M., Hegbrant J., Davenport A., Cromm K., Canaud B., Bots M.L. (2023). Physical performance tasks were linked to the PROMIS physical function metric in patients undergoing hemodialysis. J. Clin. Epidemiol..

[26] Choi S.W., Schalet B., Cook K.F., Cella D. (2014). Establishing a common metric for depressive symptoms: Linking the BDI-II, CES-D, and PHQ-9 to PROMIS depression. Psychol. Assess..

[27] Fischer H.F., Rose M. (2016). www.common-metrics.org: A web application to estimate scores from different patient-reported outcome measures on a common scale. BMC Med. Res. Methodol..

[28] Mannion A.F., Junge A., Fairbank J.C., Dvorak J., Grob D. (2006). Development of a German version of the Oswestry Disability Index. Part 1: Cross-cultural adaptation, reliability, and validity. Eur. Spine J..

[29] Fischer F., Gibbons C., Coste J., Valderas J.M., Rose M., Leplège A. (2018). Measurement invariance and general population reference values of the PROMIS Profile 29 in the UK, France, and Germany. Qual. Life Res..

[30] Fairbank J. (1995). Use of Oswestry Disability Index (ODI). Spine.

[31] Fairbank J.C. (2014). Oswestry disability index. J. Neurosurg. Spine.

[32] Cella D., Riley W., Stone A., Rothrock N., Reeve B., Yount S., Amtmann D., Bode R., Buysse D., Choi S. (2010). The Patient-Reported Outcomes Measurement Information System (PROMIS) developed and tested its first wave of adult self-reported health outcome item banks: 2005–2008. J. Clin. Epidemiol..

[33] Tang X., Schalet B.D., Hung M., Brodke D.S., Saltzman C.L., Cella D. (2021). Linking Oswestry Disability Index to the PROMIS pain interference CAT with equipercentile methods. Spine J..

[34] Cohen J. (2013). Statistical Power Analysis for the Behavioral Sciences.

[35] Kaat A.J., Schalet B.D., Rutsohn J., Jensen R.E., Cella D. (2018). Physical function metric over measure: An illustration with the Patient-Reported Outcomes Measurement Information System (PROMIS) and the Functional Assessment of Cancer Therapy (FACT). Cancer.

